# Dual role of Nrf2 signaling in hepatocellular carcinoma: promoting development, immune evasion, and therapeutic challenges

**DOI:** 10.3389/fimmu.2024.1429836

**Published:** 2024-09-02

**Authors:** Lin Gan, Wei Wang, Jinxiu Jiang, Ke Tian, Wei Liu, Zhumin Cao

**Affiliations:** ^1^ Department of Hepatobiliary Surgery, The Seventh People’s Hospital of Chongqing, Chongqing, China; ^2^ Department of Gastroenterology, The First Affiliated Hospital of Chongqing Medical and Pharmaceutical College, Chongqing, China

**Keywords:** hepatocellular carcinoma, drug resistance, molecular pathogenesis, Nrf2 dysregulation, immune evasion

## Abstract

Hepatocellular carcinoma (HCC) is the predominant form of liver cancer and ranks as the third leading cause of cancer-related mortality globally. The liver performs a wide range of tasks and is the primary organ responsible for metabolizing harmful substances and foreign compounds. Oxidative stress has a crucial role in growth and improvement of hepatocellular carcinoma (HCC). Nuclear factor erythroid 2 (1)-related factor 2 (Nrf2) is an element that regulates transcription located in the cytoplasm. It controls the balance of redox reactions by stimulating the expression of many genes that depend on antioxidant response elements. Nrf2 has contrasting functions in the normal, healthy liver and HCC. In the normal liver, Nrf2 provides advantageous benefits, while in HCC it promotes harmful effects that support the growth and survival of HCC. Continuous activation of Nrf2 has been detected in HCC and promotes its advancement and aggressiveness. In addition, Activation of Nrf2 may lead to immune evasion, weakening the immune cells’ ability to attack tumors and thereby promoting tumor development. Furthermore, chemoresistance in HCC, which is considered a form of stress response to chemotherapy medications, significantly impedes the effectiveness of HCC treatment. Stress management is typically accomplished by activating specific signal pathways and chemical variables. One important element in the creation of chemoresistance in HCC is nuclear factor-E2-related factor 2 (Nrf2). Nrf2 is a transcription factor that regulates the activation and production of a group of genes that encode proteins responsible for protecting cells from damage. This occurs through the Nrf2/ARE pathway, which is a crucial mechanism for combating oxidative stress within cells.

## 1 Introduction

Liver cancer ranks fourth in global incidence of cancer (2, 3). Primary liver malignancies are correlated with considerable clinical, economic, and psychological burdens, posing a noticeable obstacle in both developed and developing nations (4). Healthcare providers encounter hepatocellular carcinoma (HCC), which accounts for as much as 85–90% of primary liver malignancies. Variations in HCC risk factors are observed across various regions and countries. For example, chronic hepatitis B virus (HBV) infection, alcohol consumption, and hepatitis C virus (HCV) infection all contribute to the development of HCC. Risk factors, molecular subtype, and genetic landscape vary from country to country; in addition, the clinical manifestation of HCC differs by region (5, 6). Antiviral therapy and HBV vaccination are regarded as potentially effective preventative measures against the development of HCC (7, 8). Moreover, developments in the diagnosis and treatment of HCC have contributed to a substantial decline of mortality attributable to HCC by 20.3% between 1990 and 2017 (9). It should be mentioned that general cytotoxic agents such as 5-FU, adriamycin (ADM), cisplatin (DDP) and docetaxel have been advocated as chemotherapeutic agents for HCC. Furthermore, molecular target inhibitors like sorafenib have been developed to specifically target vascular endothelial growth factor receptors (VEGFR) in order to impede the development and angiogenesis of HCC. A multitude of chemotherapy strategies have been devised to address HCC. These encompass a wide range of approaches, such as traditional cytotoxic chemical therapies, gene therapy, radiation, molecular target inhibitor treatments, and medication combination techniques. that have demonstrated superior antitumor efficacy compared to monotherapy (10).

Early stages of HCC are amenable to treatment with liver resection, transplantation, and ablation, among other successful therapeutic approaches. HCC patients are frequently identified at an advanced stage, when treatment is approximately impossible, despite the progress made in diagnostic techniques (11, 12). As a result, imaging techniques that are novel should be utilized to diagnose HCC. It is recommended that patients with cirrhosis undergo screening for the development of HCC (13). Furthermore, urine, plasma, and serum can all be regarded as valuable diagnostic resources for HCC (14). A 5-year survival rate of less than 50% is observed for patients with HCC as a result of advanced stage of disease diagnosis, recurrence risk, and the metastatic character of cancer cells (15). As developing countries have a higher incidence level of HCC than developed countries, Eastern Asia and Africa account for as much as 85 percent of all HCC cases (11, 16). Genomic and epigenetic modifications are considerable contributors to the development of HCC, alongside the environmental and lifestyle factors (infection and alcohol consumption, respectively) (17).

The expression of Nrf2 is highly concentrated in HCC cells, and it is correlated with chemoresistance (18). Caused by mutations in Nfe2l2 and Keap1, the Nrf2/ARE pathway is activated, resulting in upregulation of cytoprotective proteins transcription and nuclear Nrf2 overexpression. This promotes tumorigenesis and increases the survival of tumor cells (19, 20). Moreover, chemoresistance HCC cells have an overexpression of Nrf2, which aids in the formation of chemoresistance (21–23). The expression of Nrf2 in three HCC cell lines is associated with chemoresistance to DDP (24).

The objective of recent investigations is to comprehend the signaling networks that facilitate the progression of HCC and how they interact as possible targets for therapeutic interventions (17, 25). An unfavorable prognosis is the consequence of HCC cells’ metastatic character. Migration and metastasis of HCC cells are processes facilitated by the epithelial-to-mesenchymal transition (EMT) (26, 27). For the sake of stimulating EMT-mediated migration, sphingosine-1-phosphate receptor-1 (S1PR1) is expanded in HCC (28). Controlling tumor necrosis factor (TNF) signaling in HCC is the long non-coding RNA (lncRNA) SPRY4-IT1 (29). ADAM17 raises MMP-21 expression, which accelerates the progression of HCC (30). Matrix metalloproteinases (MMPs) also contribute to the invasiveness of HCC cells. Thymoquinone upregulates miRNA-16 and miRNA-375, which inhibit the progression of HCC malignancy (31). MicroRNAs (miRNAs) also influence HCC malignancy. In order to promote the growth of HCCs and facilitate their resistance to cisplatin chemotherapy, circular RNA (circRNA)-102272 inhibits the expression of miRNA-326 (32). SIX4 has been found to enhance migration and invasion through the upregulation of c-MET and YAP1 (33). Each experiment reveals a distinct pathway that is the cause of HCC progression. On account of this, the progression of HCC cells may be mediated by a multitude of genetic and epigenetic modifications (34, 35).

The present review will focus on the role of Nrf2 in the regulation of HCC progression. Although a number of reviews have been published, they are mainly before 2022 (1, 10, 36–38) and therefore, it is required to provide an updated review in this field, as the multiple new studies have been published. Moreover, other reviews have focused on general or specific aspects such as role of Nrf2 in liver diseases (39), different cancers (40) and the function of Nrf2 in hepatitis virus-related liver tumor (41). Therefore, in the present review, the molecular pathogenesis of HCC, Nrf2 signaling and its association with tumorigenesis will be discussed. Then, the function of Nrf2 in HCC proliferation, metastasis, therapy resistance and its regulation by therapeutic compounds are discussed specifically in the current paper.

## 2 Molecular pathogenesis of hepatocellular carcinoma

The development of hepatocarcinogenesis is a multi-stage process that is characterized by variations in the pattern of gene expression that are correlated with mutations in genes that are particular to the liver. These genes, which include breast cancer 1 (BRCA1), β-catenin breast cancer 2 (BRCA2), Rb, Ras, p53, and adenomatous polyposis coli (APC),are linked to the processes of cell proliferation, progression through the cell cycle, apoptosis, and metastasis (42). The activation of growth factor modulating signaling pathways, The development of HCC is aided by various signaling pathways, including those that are involved in cell differentiation (Wnt, Hedgehog, and Notch), angiogenesis (vascular endothelial growth factor), and insulin-like growth factor (IGF), epidermal growth factor (EGF), and hepatocyte growth factor (HGF) (43).

HCC development has been linked to the disruption of signaling pathways. The development of HCC is typically associated with the dysregulation of several signaling pathways, such as Wnt/β-catenin, Ras, p14ARF/P53, p16INK4A/Rb, transforming growth factor-β (TGF-β), and PTEN/Akt (44). The Wnt/β-catenin signaling system plays a role in cellular proliferation, growth, and motility. When this route is excessively activated, it contributes to the progression of malignancy in humans (45). Fifty percent of HCC cases have been found to have an overactive Ras/mitogen-activated protein kinase (MAPK) and phosphatidylinositol 3-kinase (PI3K)-Akt kinase signaling process (46). Currently, TGFβ is believed to have a substantial impact on inhibiting cellular proliferation in the early stages and encouraging invasiveness during the last phases of HCC development (47). In spite of this, HCC develops when the JAK/STAT pathway is activated as a result of STAT- stimulated STAT inhibitor 1 (SSI-1) inactivation and subsequent activation of the pathway (48).

Recent study has shown that hepatic damage may also be influenced by Nrf2 target genes., inflammation, and hepatocarcinogenesis. HCV-induced liver cancer development can occur through the process of phosphorylation and movement of Nrf2 into the nucleus by PI3K, casein kinase 2, and MAPK. These enzymes control the expression of Nrf2-ARE genes by promoting the increase of Maf proteins (49). Nevertheless, cells infected with HBV can also stimulate an increase in the expression of the proteasomal subunit PSMB5, which is regulated by Nrf2, and lead to a decrease in the expression of the immunoproteasome subunit PSMB5i (50). In line with this, Nrf2 mRNA and protein expression was upregulated in CYP2E1-expressing HepG2 cells, and this upregulation in turn affected the expression of other downstream genes, including GCLC and HO-1 (51).

Cancer development may also be influenced by mutations and epigenetic modifications. One report revealed that 30–60% of hepatocellular carcinomas contain mutations in the telomerase reverse transcriptase (TERT) and the telomerase RNA component (TERC) (52). In turn, this contributes to the development of HCC; p53 mutation or downregulation consequently stimulates aggressive oncogenic pathways (53). Specific mutation accumulation at position 249 in p53’s seventh exon improves the proliferation of tumor cells and hinders cellular apoptosis. The dysregulation of p14/ARF, DNA damage inducible protein (GADD45), and growth arrest is associated with a high degree of chromosomal instability caused by epigenetic modifications (54). Particular instances of HCC are predominantly brought on by hypermethylation and hypomethylation at specific sites. DNA methylation in particular regions encompassing genes leads to hereditary disorder and the suppression of suppressor genes for tumors in HCC. DNA methyltransferases (DNMTs), GSTP1, E-Cadherin promoters, and Ras/Raf/ERK signaling gene pathways are the primary targets of HBV and HCV (55, 56).

## 3 Nrf2 signaling

Nuclear factor erythroid 2-related factor 2 (Nrf2) plays a vital role in protecting the body from reactive oxygen species (ROS), oxidative stress, and inflammation (57). It controls oxidative stress by affecting the activity of genes producing phase II response enzymes, including heme oxygenase-1, glutathione-s-transferase, catalase, superoxide dismutase, and NADPH quinone dehydrogenase 1 (58). The Nrf2 pathway is activated to increase and prevent stress, highlighting its crucial function in maintaining homeostasis. The Nrf2 signaling pathway is disrupted during the progression of tumor development and metastasis (59). The interactions between Nrf2 and oncogenes in tumor cells can potentially accelerate the growth of tumor cells. Recent research has demonstrated that the mammalian hepatitis B X-interacting protein (HBXIP) hinders the function of Keap1, hence promoting the activation of Nrf2 signaling. This leads to a reduction in the production of ROS in breast cancer cells. This results in heightened proliferation and spread of cancer cells (60). Nrf2 can also augment the metastatic potential of cancer cells by upregulating the expression of genes implicated in cell invasion, specifically the RhoA/ROCK pathway (61). Deubiquitinase-3 plays a crucial role in promoting resistance to chemotherapy in cancer, particularly in colon cancer cell lines. It achieves this by activating the Nrf2 pathway through inhibiting Keap1, resulting in the development of chemotherapy resistance that is dependent on Nrf2 (62). The Nrf2 pathway has been discovered to function as a defensive mechanism against the process of aging and age-related diseases (63). Protein levels of Nrf2 are regulated by three separate routes, one of which involves Keltch-like ECH-associated protein. The text refers to a protein complex called Cullin 3-ring box 1, which is related with S-phase kinase. The proteins involved are Cullin3-Rbx1/β-transducin repeat-containing protein and synoviolin/Hrd1 (64). The proteasome mechanism is linked to the degradation of Nrf2 under normal and physiological circumstances.

**Figure 1:**
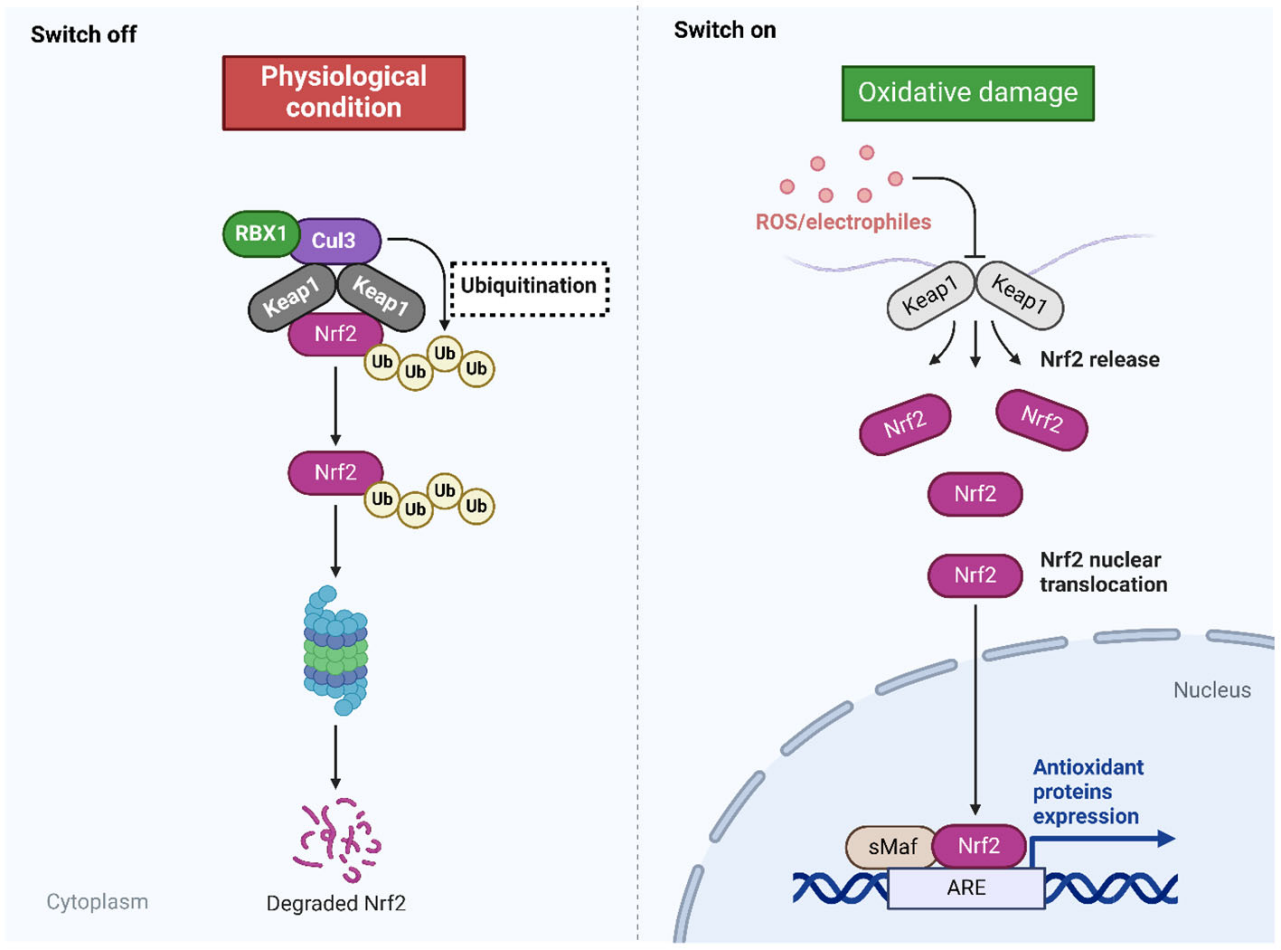
The Nrf2 signaling. The inhibition of Nrf2 is observed during physiological conditions, while the presence of oxidative damage can mediate Nrf2 signaling. In the normal condition, the Keap1 binds to Nrf2 and mediates its ubiquitination to promote its degradation by proteasome. In the oxidative damage condition, the Keap1 undergoes changes by ROS and electrophiles to improve the stability of Nrf2 for mediating its nuclear translocation binding to the genes having ARE and subsequent changes in the antioxidant defense to reduce oxidative stress. Created with BioRender.com.

Oxidative stress, characterized by elevated generation of ROS, significantly contributes to the progression of various pathological conditions, such as, but not limited to, aging, cancer, diabetes, heart failure, atherosclerosis, PD, and acute renal injury (65–67). The antioxidant defense mechanism is of paramount importance in mitigating oxidative harm. However, in situations where the burden of oxidative stress surpasses its capacity, supplementary signaling pathways are activated to compensate and bolster the effectiveness of this defense mechanism. One such pathway is the Nrf2 signaling pathway, which raises the antioxidant defense system’s capacity to counteract oxidative damage. Oxidative stress plays a pivotal role in the development of Alzheimer’s disease (AD), and two significant targets in this regard are the inhibition of mitochondrial membrane potential loss and the reduction of ROS concentrations (68, 69). Consideration may be given to targeting the Nrf2/ARE signaling pathway as a potential treatment for PD. In contrast, cancer treatment involves a distinct approach to modulating the Nrf2 signaling pathway, as it targets malignant cells to induce oxidative damage that impairs their viability and migration. The eradication of the Nrf2 signaling pathway has been linked to a reduction in breast cancer cell viability and invasion (70). The regulation of apoptotic cell death is substantially influenced by the Nrf2 signaling pathway; nuclear translocation and increased transcriptional activity of ARE have been identified as factors contributing to a reduction in apoptosis (71). In contrast, the protein kinase R-like ER kinase (PERK)/Nrf2 signaling pathway upregulates endoplasmic reticulum (ER) stress and apoptosis, thereby causing injury to cardiomyocytes (72). The significance of regulating the Nrf2 signaling pathway in mitigating inflammation is further illustrated by the effect of formononetin on the intensity of inflammation in rodents that have been exposed to methotrexate (73). Furthermore, research has provided confirmation that antioxidants that are found naturally exert a regulatory influence on the Nrf2 signaling pathway. The Nrf2 signaling pathway is significantly impacted by the principal regulators of the Nrf2 pathway, including long non-coding RNAs (lncRNAs) and microRNAs (miRs). These phytochemicals exert their influence on these regulators (57, 74–76). Endogenous antioxidants have the ability to influence these mediators, hence exerting therapeutic effects. Additionally, they possess the capacity to hinder the activation of keap1, upon which the Nrf2 signaling pathway is conditioned. This leads to the regulation of the movement of Nrf2 into the nucleus, as well as the alteration of Nrf2 mRNA expression (77–80). **Figure 1** provides an overview of Nrf2 signaling.

## 4 Nrf2 in cancer progression

Nrf2 is a transcription factor with cytoprotective properties that influences cancer in both a beneficial and detrimental manner (81, 82). Nrf2 inhibits malignant transformation by protecting both healthy and malignant cells from damage caused by free radicals; nevertheless, it provides therapy resistance once cancer has established itself (83). By exerting a selective influence on the population of tumor cells, anti-cancer therapy promotes the emergence of resistance. When it comes to chemotherapy resistance, these consist of repairing drug-induced cell injury, establishing an environment that inhibits drug release, and preventing intracellular drug exposure (84). Nrf2 is an essential factor in the development of resistance to chemotherapy as it promotes drug metabolism and efflux (85).

Proliferative capacity and colossal growth distinguish cancer cells from healthy cells; this is frequently observed in the presence of Nrf2 overactivation. The antioxidant defense function, detoxification, and other attributes of reduced GSH render it essential for cellular proliferation (86). Numerous genes involved in the synthesis of NADPH, the principal cofactor in GSH synthesis, are substantially facilitated in their transcription by Nrf2 overactivation (87). The overactivation of Nrf2 in cancer cells leads to a substantial upregulation of metabolic enzymes including G6PD, TKT, and PGD. These metabolic enzymes, which are widely expressed, facilitate the metabolism of glucose and glutamine in the PPP while also enhancing the synthesis of purines and amino acids. These processes collectively contribute to the metabolic reprogramming required for cell proliferation (88). Additionally, genes implicated in the metabolism of fatty acids and other lipids are regulated by Nrf2 (89). Furthermore, Nrf2 regulates the manifestation of microRNAs miR-1 and miR-206 in order to direct carbon flux towards PPP and the tricarboxylic acid (TCA) cycle (90).

In addition to metabolic reprogramming, Nrf2 facilitates cell proliferation. Activities of target proliferation-associated genes (Bmpr1a, Igf1, Itgb2, Jag1, and Pdgfc) are intricately linked to the regulation of the cell cycle via Nrf2 (91).The induction of G2/M phase arrests in the absence of Nrf2 indicates that Nrf2 is an essential regulator of the cell cycle through its control of inhibitory cell-cycle regulators (92). The pathways in hepatocytes that activate the phosphoinositide 3-kinase (PI3K)/protein kinase B (AKT) signaling cascade was reduced in mice lacking Nrf2. This finding suggests that by maintaining elevated levels of Nrf2, anabolic efficiency can be enhanced through the phosphorylation of the PPP, GSK3, and AKT, that sets up communication with the PI3K/AKT pathway (88). Managing the ATP substrate availability, promoting mitochondrial biogenesis, and removing mitochondrial damage are additional ways in which Nrf2 contributes to healthy mitochondrial maintenance (93). Epidermal growth factor receptor (EGFR) signaling pathway defects and oxidation of specific translational regulatory proteins were observed in pancreatic cancer cells in the absence of Nrf2. This finding suggests that Nrf2 is essential for the maintenance of cancer cells through its modulation of mRNA translation (94).

The overexpression of Nrf2 in several malignancies has led to its classification as a tumor gene (95, 96). Previous research has shown that CRC is associated with abnormally active Nrf2 signaling pathways (97, 98). On the other hand, Nrf2 expression of CRC *in vivo* has been the subject of very few investigations (96). In this respect, Lee and colleagues exhibited that the overexpression of Nrf2 accelerates the growth of colon cancer by way of the ERK and AKT signaling pathways (99). Western blotting analysis showed that Nrf2 proteins were upregulated in colorectal cancer tissues and downregulated in healthy tissues. Compared to matched normal tissues, CRC tissues exhibited higher Nrf2 expression, as revealed by IHC staining. By inhibiting cell viability and increasing cell death, short interfering RNA of Nrf2 was found to suppress SW480 cell viability. Together, these findings and the inhibition of AKT and phosphorylated extracellular signal-regulated kinase 1/2 were observed.

The various functions of Nrf2 in the progression of cancer were investigated utilizing Keap1 knockdown mice in a 2016 study. The following antioxidant genes were upregulated in mice with urethane-induced tumors of a smaller size: Glutathione reductase (Gsr), Ppargc1A,Glutathione peroxidase 2 (Gpx2), Glutathione-S-transferase a4 (Gsta4), and Catalase (Cat). Nevertheless, the tumors demonstrated a greater degree of aggressiveness when transplanted into nude rodents (100). Moreover, in cases of traditional Hodgkin lymphoma, it was discovered that, in contrast to more severe manifestations of the disease, a reduced prevalence of the condition is associated with increased Nrf2 expression (101). This means that the disease is more confined in its progression. When Nrf2 is overexpressed in glioblastoma cells, it causes an increase in the number of cells that proliferate and undergo oncogenic transformation (102).

Elevated proliferation and inhibition of apoptosis are outcomes of the Nrf2 signaling pathway in cervical cancer (103). Angiogenesis is facilitated by Nrf2 activation in breast cancer, which concurrently inhibits the expression of estrogen-related receptor α and induces upregulation of Rho and Focal adhesion kinase 1, modulator of volume-regulated anion channel current 1, and Rho-associated coiled-coil-containing protein kinase 1 are its downstream proteins. Furthermore, Nrf2 promotes enhanced protein stability by means of a direct interaction with breast cancer type 1 susceptibility protein (BRCA1). Estrogen reinstates Nrf2 activation in the absence of BRCA expression, resulting in protection of mammary gland cells and a reduction in *in vitro* ROS production (104). Through interacting with Nrf2 in the promoter region of this gene, we can decrease the expression of the vascular cell adhesion molecule (VCAM). Pro-oxidants 15-lipoxygenase (15-LOX) and exogenous antioxidants phospholipid hydroperoxide glutathione peroxidase (PHGPx) were effective (105).

Arrest defective 1 (ARD1) mediates cell functions like homeostasis, migration, differentiation, proliferation, and tumorigenesis (106). Elevated expression is linked to cancer, poor prognosis, and suppresses tumorigenesis by inhibiting mTOR signaling, as reported in various studies (107, 108). In this regard, Fang and colleagues demonstrated that through direct contact, ARD1 stabilizes NRF2 and promotes the growth of colon cancer (106). The study found that ARD1 knockdown in human colon cancer cell lines reduced NRF2 protein levels without affecting its mRNA expression. However, silencing NRF2 did not alter ARD1 protein expression. ARD1 overexpression increased NRF2 acetylation levels, and mutant forms of ARD1 with defective acetyltransferase activity reduced NRF2 stability.

The growth and dissemination of cancer cells are supported by Nrf2. An increased expression of Bcl-xL (B-cell lymphoma extra-large) and MMP9 (matrix metallopeptidase 9) in HCC has been found to correlate positively with the activation of the Nrf2 signaling pathway, according to research. By causing the basal membrane to be breached, the metalloproteinase MMP-9 contributes to cancer invasion. Associated with anti-apoptotic activity, Bcl-xL is a factor that inhibits cell death (apoptosis) (109). Nrf2 upregulated the expression of MMP9 in glioma cells (110). The Nrf2 signaling pathway reduced apoptosis by over activating the anti-apoptotic protein, Bcl-2. The promoter region of Bcl-2 contains an ARE region located on the reverse strands at positions −3148 and −3140 (111).

Lung mucoepidermoid carcinoma with overexpressed HMOX1 was found to be linked to reduction of Cyclin D1 (CCND1) and CCND2, two proteins involved in cell cycle progression, as well as the activation of the transcription of Cyclin-dependent kinase inhibitor 1C (CDKN1C) and cell-cycle arrest proteins Gastrin (GAS). Using small interfering RNA (siRNA), the expression of invasion promoters (MMP-9, MMP-1, and MMP-12), pro-inflammatory cytokines (IL-6, IL-1β, and TNFα), and the pro-angiogenic factor VEGFA was reduced by silencing the HO-1 gene (112). The anti-inflammatory cytokineIL-11 is associated with Nrf2 overexpression, which is a noteworthy finding in breast and lung cancer tissues. This particular interleukin belongs to the family of IL-6 and is linked to the development of epithelial malignancies, such as breast cancer and gastric, that are caused by the inflammasome (113). The Nrf2 antioxidant activity contributes to the apoptosis of regulatory T lymphocytes (T regs), which opposes the positive effects of PD-L1 anticancer therapy (114).

In addition to preventing cancer cell resistance to chemotherapy, the Nrf2 signaling pathway is vital for preserving cells and tissues against oxidative injury (115). Preventing the severe cytotoxicity that chemotherapy agents can inflict on healthy cells is a critical aspect of minimizing their adverse effects. In contrast, Nrf2 activation may also result in chemoresistance by forming a barrier against cancer cell injury (116, 117). Chemoresistance in malignancy has been linked to Nrf2 signaling, with activation of this pathway inducing resistance to chemotherapy. Chemoresistance is inversely proportional to the function of Nrf2 signaling inhibition. Suppressing the production of ROS in cancer cells, thioredoxin reductase 1 (TR1) is stimulated subsequent to activation, thereby inducing resistance to 5-fluorouracil (118). Chemoresistance is induced when tumor-promoting factors such as Progestin and AdipoQ Receptor 4 impede Nrf2 degradation and increase its stability (119). It appears that Nrf2 can promote the lineage of tumor-initiating cells, which in turn facilitates chemoresistance (120, 121). Chemoresistance can be achieved when Keap1 is proteasomally degraded by the p53 through the induction of Nrf2/ARE signaling, which promotes proliferation and inhibits apoptosis (122). Chemoresistance is induced and glutamine metabolism increases in response to Nrf2 activation; this is associated with a poor prognosis for cancer patients (123). Anti-cancer chemoresistance and growth are promoted by Nrf2 activation, whereas inhibition of its activity increases chemotherapy sensitivity (124).

There is a close relationship between Nrf2 and the immune microenvironment. The immune microenvironment refers to the cellular, cytokine, and molecular signaling factors surrounding tumors, which are crucial for regulating tumor growth, metastasis, and treatment responses (125, 126). Nrf2 plays a significant role in the immune microenvironment, influencing the interaction between immune cells and tumor cells, as well as the regulation of immune responses. Nrf2 regulates the expression of antioxidant enzymes, thereby reducing the oxidative stress levels in the immune microenvironment and alleviating oxidative damage to immune cells and tumor cells (127). Additionally, Nrf2 can also inhibit the occurrence of inflammatory reactions, thereby decreasing the levels of inflammation in the immune microenvironment. In addition, Nrf2 can influence the production of multiple cytokines, such as regulating tumor necrosis factor-alpha (TNF-α), interleukin-6 (IL-6), and others (128, 129). These cytokines play crucial roles in the immune microenvironment, impacting the activity of immune cells and the status of the tumor microenvironment. Moreover, the status of the immune microenvironment directly influences the recognition and clearance of tumor cells by immune cells. Activation of Nrf2 may lead to immune evasion, weakening the immune cells’ ability to attack tumors and thereby promoting tumor development. In general, Nrf2 regulates various aspects of the immune microenvironment, including oxidative status, inflammation levels, and cytokine production, affecting the interaction between immune cells and tumor cells and the regulation of immune responses. In-depth research on the role of Nrf2 in the immune microenvironment contributes to understanding the mechanisms of tumor immune evasion and provides new targets and strategies for tumor therapy.

The function of Nrf2 in the regulation of tumorigenesis has been more important in the recent years. Nr2 can mediate therapy resistance in cancer, as the downregulation of Nr2 by human papillomavirus has been shown to enhance sensitivity to radiotherapy and chemotherapy (130). In order to increase the potential in tumor suppression the co-application of Nr2 and GPX4 inhibitors has been suggested to exert synergistic impact in ovarian cancer therapy (131). Notably, the induction of endoplasmic reticulum (ER) stress has been a promising strategy in reducing the tumorigenesis. However, SMURF1 increases KEAP1 degradation to induce Nrf2 axis for reducing ER stress (132) and this can be further evaluated in the treatment of cancer. The downregulation of Nr2 by brusatol has been suggested to decrease growth and metastasis of thyroid cancer (133). The similar approach has been also shown in colorectal tumor that downregulation of Nr2 by triptolide can impair the growth and metastasis (134). As another anti-cancer agent, dihydrotanshinone I can increase Nr2 degradation by Keap1 and it impairs Nr2 phosphorylation by PKC to reduce proliferation of gallbladder tumor (135). Furthermore, the upregulation of Nr2 and downregulation of KEAP1 mediate the unfavorable prognosis in breast cancer (136). The metabolism of tumor cells can be affected by Nr2 axis. Noteworthy, B7-H3 can stimulate Akt/Nr2 axis in enhancing glutathione metabolism, elevating stemness in gastric tumor (137). One of the interesting points is the regulation of cell death mechanisms by Nr2 in human cancers. Quercetin is able to bind to SLC1A5, disrupting the nuclear transfer of Nr2 for downregulating GPX4, causing ferroptosis in gastric tumor (138). In line with this, CPT1B can increase Nr2 levels through KEAP1 inhibition to improve redox homeostasis and reduce ferroptosis in pancreatic tumor, causing gemcitabine resistance (139). Isoorientin is able to downregulate SIRT6 for suppressing Nrf2/GPX4 axis, causing ferroptosis in lung tumor and overcoming chemoresistance (140). The increase in stabilization of Nr2 by DPP9 can diminish ferroptosis in accelerating sorafenib resistance in renal cancer (141). Moreover, there is interaction between Nr2 and epigenetic factors such as miR-34 and miR-29b-3p in the regulation of tumorigenesis (142, 143). Furthermore, the induction of Nr2 in the tumor-associated macrophages can induce immune resistance in cancer (144). Therefore, increasing evidences highlight the function of Nr2 in the regulation of tumorigenesis (145–150) and therefore, therapeutic targeting of Nr2 can reverse cancer progression.

## 5 Nrf2 activation in hepatocarcinogenesis

In the last thirty years, researchers have uncovered the Janus aspect of cellular oxidants, which possesses both beneficial and detrimental biological effects (1, 151). Maintaining their function as reaction substrates in metabolic processes and second mediators in redox-sensitive signal transduction pathways, cells are in a constant state of effort to prevent oxidative stress (151, 152). This includes autophagy, which may have multiple or dual functions in cancer metabolism. The Keap1-Nrf2 pathway serves as a vital cellular detector for electrophilic substances, stimulating adaptive gene responses to safeguard cells against ROS and oxidative stress. This system helps maintain redox homeostasis and signaling functions (153–156). The integrity of the liver is essential for optimum liver function, as it plays a critical role in protecting cells (157). Nrf2, a vital gene in the CNC-basic region leucine zipper family, is downregulated by the interaction protein Keap1, an adaptor of the Cul3-ubiquitin E3 ligase complex. This complex is responsible for the degradation of Nrf2 in cells that are not stimulated or in a resting state (158, 159).

When there are high levels of ROS/NOS/electrophiles, Nrf2 becomes stable and builds up in the cytosol because Keap1 is deactivated. It then moves into the nucleus and helps activate gene transcription by forming a complex with small Maf proteins (sMaf) and binding to specific sequences (ARE/ERE) in the genes responsible for producing proteins that balance redox, detoxify, transport drugs, regulate metabolism, and aid in β-oxidation (160, 161). Modulating the amounts of Nrf2 protein can either enhance or safeguard cells against oxidative stress-induced cell death. Nevertheless, the involvement of Nrf2 in the regulation of homeostatic response and the development of human diseases seems to be more intricate than initially anticipated. The stimulation of the Nrf2 pathway in cells and tissues has been found to have a U-shaped effect. Due to its impact on several metabolic pathways, it is challenging to determine a specific role for this transcription factor in physiological and pathological responses (154). Excessive Nrf2 activity can harm cells and tissues, resulting in situations of allostatic stress and poor cellular metabolism. These adverse consequences can contribute to the development of degenerative lesions and rapid aging of tissues, which are commonly seen in advanced chronic conditions such as diabetes, chronic kidney disease, and metastasized cancer.

Several ways have been devised to specifically enhance the beneficial benefits of Nrf2 transcriptional activity in the field of anti-aging therapy, while preventing any drawbacks that may arise from excessive stimulation. These tactics involve examining the impact of transcription on genes related to cytoprotection and detoxification, namely those regulated by Nrf2. Additionally, they explore the potential chemopreventive effects of various natural and synthetic substances, such as dietary antioxidants, plant phenolics, triterpenoids, and selenium compounds (154, 162–164). On the contrary, the expression of Nrf2-dependent genes involved in chemoprevention and cytoprotection may play a role in the development of chronic and age-related diseases, such as liver disorders and different types of human malignancies (154, 155, 165, 166). Research indicates that mutations in the Keap1/Nrf2 pathway or imbalanced regulation of this system contribute to the development of tumors in people and facilitate the advancement of cancer by continuously activating Nrf2 and altering cell metabolism (83). Reducing the expression of Nrf2 makes cancer cells more susceptible to oxidative stress and chemotherapy (167).

The presence of Nrf2 mutations can cause hepatocarcinogenesis (168, 169). As ubiquitin E3 ligase, TRIM21 has been shown to increase KEAP1 suppression by p62 in increasing Nrf2 levels to support against cell death and ROS, enhancing hepatocarcinogenesis (170). During HCC development, the nuclear transfer and localization of Nrf2, MAF and KEAP1 increase. Moreover, co-localization of Nrf2 and KEAP1 is high in the cell nuclei of HCC neoplastic nodules (171).

Nrf2 has a significant impact on the development of liver cancer, as shown in experiments on rats that have been genetically altered to study drug toxicity. A study found that animals with a genetic modification that inhibits the Nrf2 protein were more prone to liver blood vessel changes, resulting in a reduction in drug-induced liver damage (172). An alternative manifestation of Cyp2e1 protein was detected in Nrf2−/− animals, resulting in vascular alterations and reduced hepatotoxicity of acetaminophen (1). The modification of the Keap1 gene has been employed to induce chronic activation of Nrf2 and enhance the susceptibility to developing HCC in mice treated with carcinogenic chemicals. The genetic removal of this Nrf2 inhibitory protein disrupts the process that protects hepatocytes from drug-induced toxicity and impairs cell viability by interfering with the cell’s ability to eliminate protein aggregates (173). In a recent study conducted by Ngo and his colleagues, it was found that overactivation of Nrf2 is necessary for the progression of HCC produced by diethylnitrosamine (DEN) in mice. The increased activity of Nrf2 in the tumor was due to changes in its ability to bind to the natural inhibitor Keap1 (169). These investigations emphasize the intricate significance of Nrf2 activation in the development of liver cancer and identify a significant role of Keap1 in this process. The Nrf2-Keap1 pathway plays a significant role in HCC by activating genes that control several aspects of liver function. This suggests potential chances for creating ways to prevent and treat HCC using chemicals (1).

## 6 Nrf2 in hepatocellular carcinoma proliferation and metastasis

Respiratory oxidative stress (ROS) caused by endogenous metabolites or pollutants may cause mutations in important genes. Unusual activation of Nrf2 may result from somatic mutations that disrupt the binding between Keap1 and Nrf2 or Keap1 and Nrf2 (36). Driver mutations can promote cancer by providing a selective growth advantage. Thirty candidate driver genes were identified in 503 genomes of liver cancer (174, 175). In HCC, Nrf2 mutations occur more frequently (5.1%) than Keap1 mutations (3.2%) (176). As evidenced by the presence of Keap1 and Nrf2 mutations in the advanced stages of human liver carcinogenesis, HCC develops belatedly (177). On the contrary, Nrf2 mutations manifest themselves early on in the resistant hepatocyte model, ultimately resulting in the development of cancer (168). The majority of mutations observed in HCC-associated Nrf2 occur in the high affinity ETGE motif and the low affinity DLG motif (168, 178). On the other hand, changes to the DLG motif can stimulate Nrf2 activation. Keap1 mutations resulting in function loss result in the sustained activation of Nrf2. According to data from next-generation sequencing, 6% of HCC are the outcome of oxidative stress brought on by ROS or mutations. The nuclear translocation of Nrf2 is enhanced when Keap1 and Nrf2 are dissociated by oxidative stress; this process regulates the expression of antioxidant genes (179).

There exists a correlation between Nrf2 expression and both cellular survival and clinicopathological factors in HCC (109). The correlation between increased Nrf2 expression and tumor size, metastasis, and differentiation was observed in HCC. The invasion and proliferation of HCC cells were further confirmed by the presence of matrixmetallaproteinase-9, and Bcl-xL in addition to Nrf2 expression. Therefore, Nrf2 expression may serve as a self-referential indicator in the context of HCC. An intriguing finding was reported regarding Hepa-1 and HepG2 cells, in which Bcl-xL expression was mediated by Nrf2 via AREs located in the Bcl-xL promoter area (180). The induction of Bcl-xL expression by Nrf2 in both cell lines facilitated drug resistance and survival while inhibiting apoptosis. Evidence of 8-hydroxyguanosine (8-OHdG) damage and elevated Nrf2 expression were identified in patients with HCC (181). This elevated 8-OHdG concentration was uncovered to be linked with poor survival. Comparable observations were also detected in HepG2 cells subsequent to H2O2 exposure.

In their investigation, Xi and colleagues illustrated that Nrf2 promotes the development of HCC by means of metabolic and epigenetic regulatory networks mediated by acetyl-CoA (182). The study reveals that Nrf2 ablation in a mouse model of HCC disrupts multiple metabolic pathways, reducing acetyl-CoA generation and suppressing histone acetylation in tumors but not in normal tissue. This results in a low glucose-dependent regulatory function of Nrf2, which is demolished under energy refeeding. The findings suggest that Nrf2’s role in acetyl-CoA generation and its antioxidative stress response is crucial for HCC progression.

Moreover, chronic exposure to the non-genotoxic hepatocarcinogens piperonyl butoxide (PBO) and pentachlorophenol (PCP) induced oxidative stress in Nrf2-deficient mice (183). Oxidative stress has the potential to promote the growth and advancement of preneoplastic lesions into malignant neoplasms. This finding suggests that dysregulation of Nrf2 raises the susceptibility to carcinogenesis induced by environmentally benign carcinogens lacking genotoxicity.

In another investigation, Liu and colleagues demonstrated that by focusing on the Keap1-Nrf2 pathway, TRIM25 enhances hepatocellular carcinoma cell survival and proliferation (184). TRIM25 is induced by ER stress, promoting oxidative stress, ER-associated degradation, and reducing IRE1 signaling. Depletion leads to ER stress and tumor cell growth. TRIM25 targets Keap1, activating Nrf2, bolstering anti-oxidant defense and cell survival. High TRIM25 expression correlates with poor patient survival in HCC, and positively with Nrf2 expression.

Further, miR-200a expands HCC cell proliferation via targeting the Nrf2 pathway’s Keap1. When miR-200a mimics are transfected into HepG2 and FaO and RH HCC cells from rats, it prevents the expression of Keap1 (185). Elevated cell proliferation may be a result of Nrf2 upregulating its own target genes when miR-200a downregulates Keap1. Additionally, Yang and colleagues illustrated that in order to impede the advancement of HCC, the phytollin I induced ferroptosis by stimulating mitochondrial dysfunction via the Nrf2/HO-1/GPX4 axis (186). Based on consequences, PPI inhibited HCC cell proliferation, invasion, and metastasis by increasing reactive oxygen species, promoting Fe2+ accumulation, depleting GSH, and suppressing xCT and GPX4 expression. This induction was linked to PPI binding to Nrf2, HO-1, and GPX4 proteins, modulating the Nrf2/HO-1/GPX4 antioxidant axis. PPI also caused mitochondrial structural damage and decreased MMP. *In vivo*, PPI inhibited Nrf2/HO-1/GPX4 axis-induced ferroptosis, similar to sorafenib’s effects.

Hepatocyte transformation of HCC progenitor cells can occur as a consequence of cellular oxidative stress induced by the accumulation of ROS in hepatocytes exposed to chemical carcinogens (187). Protection is provided throughout this process by a brief activation of Nrf2.; conversely, an ongoing Nrf2 activation can facilitate the progression of cancer. The maintenance of Nrf2 activation By activating Nrf2 with antioxidants, carcinogenesis will be accelerated. Inhibitors of Nrf2, which have the potential to impede the advancement of chronic liver injury to hepatocellular carcinoma, thus constitute a potentially fruitful therapeutic approach.

Another study conducted by Zheng and colleagues revealed that in HCC, pseudohypoxia is caused by overactive NRF2 through the stabilization of HIF-1α (188). The study found that protein expression of NRF2 was unaffected by inhibiting HIF-1α in HepG2 human hepatoma cells. However, the nuclear accumulation of HIF-1α was decreased without any change in its mRNA expression when NRF2 was knocked down. In the case of hepatocarcinogenesis induced by diethylnitrosamine, NRF2 expression was elevated and upregulated, in contrast to mice deficient in Nrf2. NRF2 and HIF-1α were physically interacted with, and their upregulation was discovered in tumor samples taken from patients with HCC.

STC2 has been shown to function as biomarker in HCC and is upregulated in this tumor. Notably, the expression of STC2 decreases in Nrf2-deficient cells (189), highlighting the function of Nrf2 as biomarker. Nrf2 can participate in controlling the metastasis of HCC cells through induction of EMT (190). Briefly, EMT is characterized with N-cadherin and vimentin upregulation, and E-cadherin downregulation. The stimulation of EMT accelerates cancer metastasis and mediates chemoresistance (191, 192). NOP16 stimulates EMT to enhance HCC metastasis, while it knockdown induces KEAP1/Nrf2 axis in metastasis suppression (190). Moreover, the inhibition of ROS/Nrf2/Notch axis by phellodendronoside A has been suggested to accelerate apoptosis in HCC (193). One of the mechanisms in improving tumorigenesis of HCC by Nrf2 is maybe related to impairing the function of immune system. Notably, mutation of Nrf2 can downregulate STING to promote immune escape in HCC (194). In the recent years, immune evasion has been obvious in cancers and nanoparticles have been suggested in cancer immunotherapy (195). Therefore, the role of Nrf2 in immune evasion of HCC and its regulation by nanoparticles in HCC therapy should be further highlighted. Another part is also related to increase in the stemness of HCC cells. Noteworthy, fine particulate matter (PM2.5) has been suggested to induce ROS/Nrf2/Keap1 axis for triggering autophagy in enhancing HCC progression and stem cell-like features (196).

**Figure 2:**
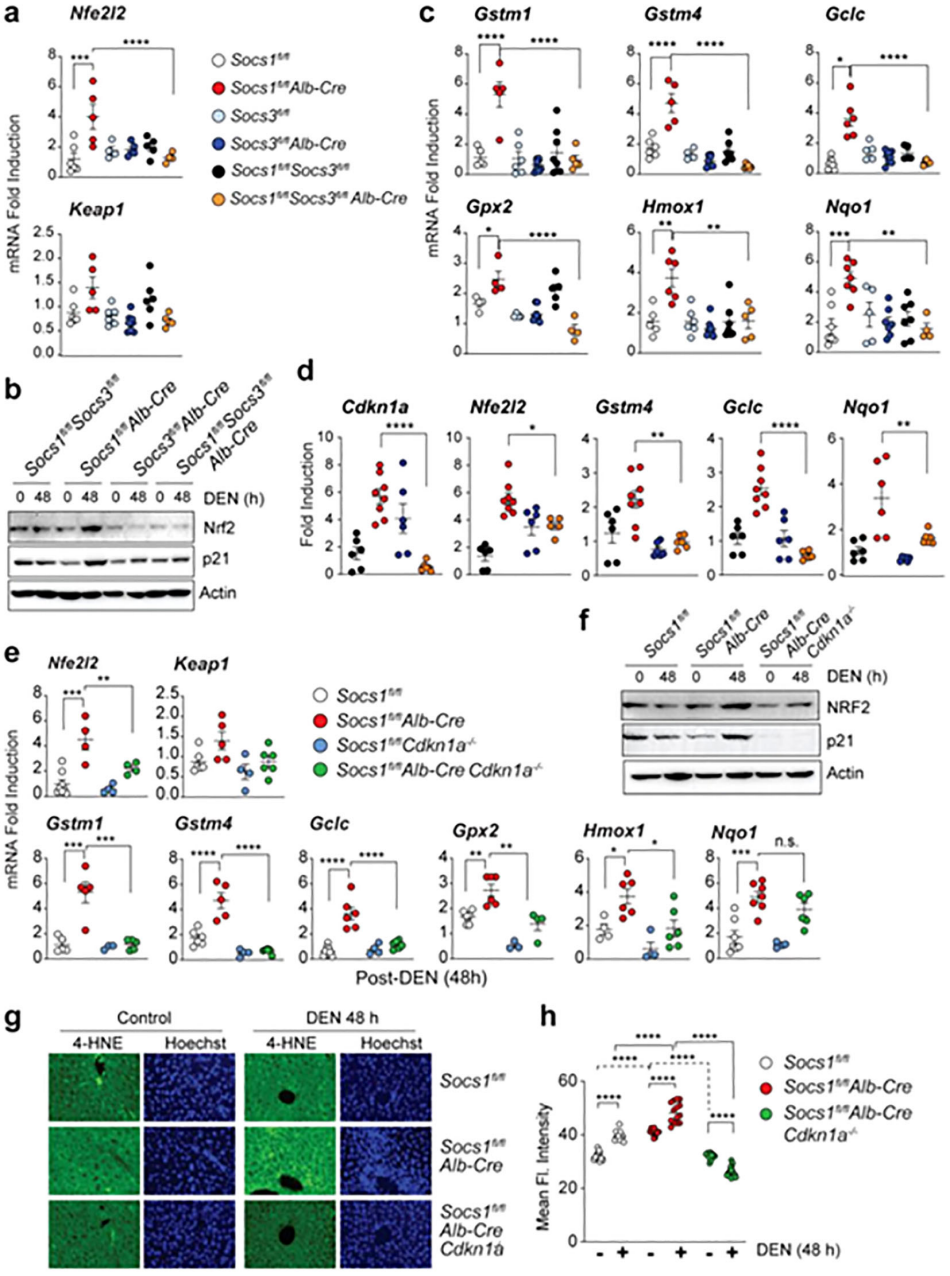
The upregulation of Nrf2 can occur upon the loss of SOCS1. **(A, B)** Induction of the Nfe2l2 gene **(A)** and NRF2 protein **(B)** in the liver tissues of mice lacking SOCS1, SOCS3, or both in hepatocytes 48 hours after DEN treatment. Cumulative data from 3–8 mice per group are shown in **(A)**. For **(B)**, representative data from more than two experiments are shown. **(C)** Induction of NRF2 target genes in mice lacking SOCS1, SOCS3, or both in hepatocytes 48 hours after DEN treatment (n = 3–8 mice per group). **(D)** Expression of Cdkn1a, Nfe2l2, and NRF2 target genes in microscopically dissected DEN-induced HCC tumor nodules resected from the indicated genotypes of mice (n = 5–8 mice per group). **(E)** DEN-induced Nfe2l2 and NRF2 target gene expression in SOCS1-deficient mice lacking CDKN1A. Gene expression data for Socs1^fl/fl and Socs1^fl/flAlb-Cre are duplicated from **(A, C)** for comparison. **(F)** Immunoblot analysis of NRF2, p21, and actin in the livers of DEN-treated mice of the indicated genotypes (n > 3). **(G)** 4-HNE staining for lipid peroxidation in DEN-treated mice livers (40× magnification). Representative images from more than 3–4 mice per group are shown. **(H)** Quantification of 4-HNE staining from 3–4 mice per group. Reprinted from MDPI (197). *p<0.05, **p<0.01, ***p<0.001, ****p<0.0001. n.s, Not significant.

Moreover, the expression of CDKN1A in the liver is enhanced in patients with SOCS1 deficiency, who are more likely to develop HCC. Severity of disease is associated with high CDKN1A expression in several malignancies. In this regard Ilangumaran and co-workers exhibited that hepatocellular carcinoma advances in SOCS1 deficiency through SOCS3-Dependent CDKN1A induction and NRF2 activation (**Figure 2**) (197).

**Figure 3:**
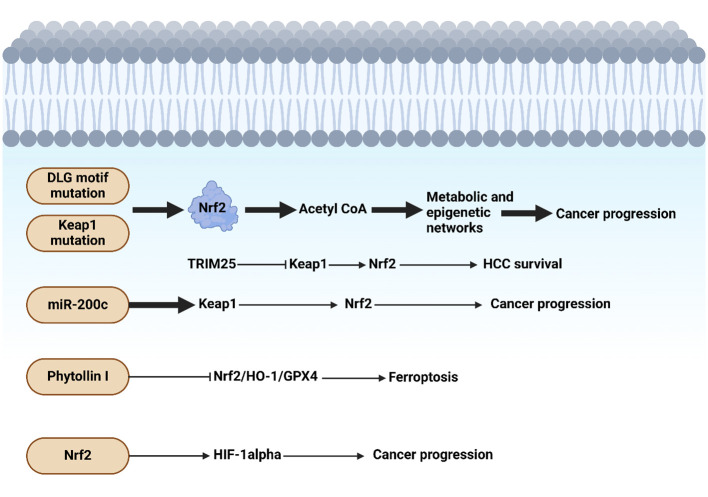
Nrf2 function in hepatocellular carcinoma progression. The presence of mutations in DLG motif and Keap1 can induce Nrf2 that through acetyl-CoA, it can mediate metabolic and epigenetic alterations to enhance cancer progression. Moreover, miR-200c downregulates Keap1 to upregulate Nrf2 in tumorigenesis. Phytollin I stimulates ferroptosis through suppression of Nrf2/HO-1/GPX4 axis. The upregulation of HIF by Nrf2 can accelerate carcinogenesis. Created with BioRender.com.

The study shows liver cells lacking SOCS1 increase SOCS3 expression, which in turn promotes p53 activation and Cdkn1a induction. This causes SOCS1-deficient livers to experience an increase in the induction of NRF2 and its target genes after DEN therapy. The growth of DEN-induced hepatocarcinogenesis is reduced in SOCS1-deficient animals when they lose SOCS3, but tumor incidence is unaffected. Patients with HCC who do not have enough SOCS1 but have high levels of SOCS3 may benefit from targeting the NRF2 pathway. Kudo and collaborators revealed that the progression of liver cancer is accelerated by autophagy, oxidative phosphorylation, and NRF2 when PKCλ/ι is lost (198). The study found that oxidative phosphorylation and autophagy are caused by the inactivation of protein kinase C (PKC) λ/ζ in hepatocytes., cause reactive oxygen species (ROS) production and, via both cell-autonomous and non-autonomous pathways, propel hepatocellular carcinoma (HCC). Levels of PKCλ/ι have been found to have a negative correlation with the histological tumor grade of HCC, confirming its role as a tumor suppressor in liver cancer. **Figure 3** shows the function of Nrf2 in the progression of HCC.

## 7 Nrf2 in hepatocellular carcinoma drug resistance and its modulation by therapeutic compounds

Resistance to chemotherapy is a common risk in the treatment of HCC. Chemoresizable HCC cells may be sensitized by phytochemicals and molecules. Apigenin (4′,5,7-trihydroxyflavone) is an inherent bioflavonoid with demonstrated efficacy in combating a wide range of malignancies. Apigenin was found to increase the delicate nature of doxorubicin-resistant HCC (BEL-7402/ADM) at concentrations that were noninvasive (23). The amount of adriamycin (ADM) within the cells was elevated in BEL-7402/ADM cells, apigenin induced an increase in cytotoxicity. By activating Nrf2, the PI3K/Akt pathway confers chemoresistance to cancer cells. Apigenin functions as a potent adjuvant sensitizer in conjunction with ADM by impeding the PI3K/Akt and its subsequent Nrf2 pathways. Consequently, this induces ADM sensitization in the BEL-7402/ADM cells. Chrysin (5,7-dihydroxyflavone), an additional naturally occurring flavonoid, induces doxorubicin sensitivity in ADM-resistant BEL-7402/ADM cells by inhibiting the PI3K-Akt and ERK pathways (199). By ultimately preventing these pathways, Nrf2 expression is suppressed and medication-resistant characteristic in BEL-7402/ADM cells.

Moreover, Elkateb and colleagues demonstrated that Camptothecin enhances the sensitivity of Hepatocellular carcinoma cells undergo sorafenib-induced ferroptosis through the suppression of Nrf2 (200). The study found that sorafenib and CPT have a strong synergy, enhancing lipid peroxidation and iron levels while reducing total antioxidant capacity and enzyme activity. It also decreases the expression of Nrf2 and SLC7A11, which is crucial for decreasing HCC cell resistance to sorafenib. Inhibiting Nrf2 with CPT enhances sorafenib sensitivity and resistance by promoting the ferroptosis mechanism of action, thereby decreasing resistance.

Furthermore, the main medicine licensed by the food and medication delivery system for epilepsy is valproic acid (VPA), a short-chain branching fatty acid and an inhibitor of histone deacetylase (201, 202). The proton beam caused apoptosis in Hep3B cells due to the ROS and DNA damage received by VPA. When combined with proton beam irradiation, VPA produces a synergistic effect (203). Hep3B cells and xenograft models of Hep3B tumors both exhibited downregulated Nrf2 expression. By focusing on the Nrf2 signaling pathway, VPA serves as a radiosensitizer in HCC. Researchers found that phytochemicals and other compounds may be able to sensitize chemoresistant HCC by inhibiting Nrf2. The sensitivity of HCC cells to ferroptosis can be enhanced through regulation of Nrf2. Camptothecin downregulates Nrf2 top enhance sorafenib-mediated ferroptosis in HCC therapy (200).

Furthermore, the expression of miR-141 confers HCC with resistance against 5-fluorouracil (5-FU). 5-FU triggers apoptosis in cancer cells by reducing the levels of antioxidants, making it an effective chemotherapeutic agent for treating many types of malignancies (204). The resistance is caused by an upregulation of miR-141 expression. It was found that HuH7/5-FU cells, SMMC-7721/5-FU, and HepG2/5-FU had greater expression of miR-141 compared to their original counterparts (205). miR-141 suppressed the process of programmed cell death caused by 5-FU in HCC. miR-141 specifically binds to the 3′-UTR of Keap1 mRNA, resulting in a reduction in the amounts of both Keap1 mRNA and protein. MiR-141 downregulates Keap1 expression, leading to the activation of the Nrf2 pathway, which ultimately results in HCC being resistant to 5-FU. In addition, Zhou and colleagues represented that miR-144 targets the Nrf2-dependent antioxidant system, which hepatocellular carcinoma cell lines use to regain chemoresistance (21). The study discovered that miR-144 was markedly down-regulated in Bel-7402/5-FU cells when compared to parental Bel-7402 cells, and it was also dramatically lowered in HCC cell lines. Enhanced cytotoxicity and cell death in 5-FU-induced cells were associated with this Nrf2 down-regulation. MiR-144 lowered Nrf2 levels, hindered the transcription of the Nrf2-dependent HO-1 gene, and accelerated nuclear factor erythroid-2-related factor-2 mRNA degradation, all of which contributed to 5-FU sensibilization. Re-expressing Nrf2 reduced the chemosensibilization effect of miR-144 to a lesser extent.

In addition, many fruits and herbs contain (UA, the triterpenoid is a lipophilic pentacyclic compound that gives apples and other fruits a smooth waxy appearance (206). With regard to HepG2/DDP cells that have developed resistance to cisplatin, UA reversed the resistance to cisplatin (22). One main way UA works is by blocking Nrf2 signaling. Therefore, UA can be used to treat cisplatin-resistant chemoresistant HCC by acting as a natural adjuvant sensitizer. Besides, Nrf2 plays a crucial role in regulating glucose metabolism, This is crucial for the development of tumors (207). It is activated by a redox-dependent mechanism that inhibits miR-1 and miR-206 expression. In addition, epigenetic processes like acetylation and DNA methylation control their expression. In tumors, persistent Nrf2 activation inhibits HDAC4 activation, attenuating the expression of miR-1 and miR-206. This attenuation targets the pentose phosphate pathway genes, regulating glucose metabolism and ultimately leading to tumor growth progression (90, 208). Moreover, the multi-kinase inhibitor sorafenib, better known by its brand name Nexavar, is an effective anti-cancer medication, especially in cases of advanced HCC (209). Sorafenib stops the invasion and proliferation of cells. One major issue with HCC is the development of chemoresistance to 5-FU. As a result of suppressing Nrf2, sorafenib reduces 5-FU resistance in BEL-7402/5-FU cells (210). This proposes that sorafenib may have potential as a Nrf2 inhibitor for HCC.

A significant component of herbal tea, 2′,4′-Dihydroxy-6′-methoxy-3′,5′-dimethylchalcone (DMC) is a chalcone chemical discovered in the buds of Cleistocalyx operculatus (211). DMC has been shown to have hepatoprotective effects in mice (212), and anti-tumor activity in SMMC7721 HCC cells (213). In BEL-7402/5-FU cells, by decreasing intracellular GST activity and GSH concentration, DMC was discovered to block medication efflux (214). Furthermore, DMC acts as a Nrf2 inhibitor, suppressing the expression of Nrf2 and subsequently downregulating the expression of glutamate-cysteine ligase catalytic subunit (GCLC) and glutamate-cysteine ligase modifier subunit (GCLM).

This pigment is a natural anthocyanidin that is polyphenolic., pelargonidin can be found in high concentrations in radishes and berries (215). A decrease in citrinin-induced cytotoxicity in HepG2 cells was seen after pre-treatment with pelargonidin chloride (PC). Through the activation of the Keap1-Nrf2 pathway, PC was found to increase the number of detoxification enzymes as well as their activity, as revealed by mechanistic insights. Moreover, the compound known as rebaudioside (13-[(2-O-β-d-glucopyranosyl-3-O-β-d-glucopyranosyl-β-d-gluco pyra nosyl) hydroxide])The steviol diterpene glycosidic component known as kaur-16-en-18-oic acid β-d-glucopyranosyl ester is extracted from the leaves of Stevia rebaudiana Bertoni (216). Rebaudioside, often known as Reb A, is a natural sweetener that has a sweetness that is between 250 and 450 times higher than that of sucrose. It is utilized as a sugar substitute that does not include calories in a number of nations (217). In a recent review, number of toxicological and pharmacological findings of Reb A were examined (218). Reb A was found to exhibit antioxidant action in HepG2 cells, where it was able to mitigate the oxidative damage caused by carbon tetrachloride (CCl4) (219). The activation of Nrf2 that was induced by Reb A was observed in HepG2 cells that had been treated with CCl4. HO-1 and NQO1 expression, followed by Nrf2 activation were responsible for the recovery of oxidative damage. It was through the increase of JNK, ERK, MAPK, and PKCϵ signaling that this boost of Nrf2 activation took place.

Crotonaldehyde, an exceptionally reactive α, β-unsaturated aldehyde, is a prevalent component in cigarette smoke, as well as in meat, vegetables, bread, cheese, and fruits (220). Crotonaldehyde induced HO-1 expression in HepG2 cells via the translocation and activation of Nrf2 into the nucleus (221). The Nrf2 pathway was finally instigated through the p38 mitogen-activated protein kinase pathway and protein kinase C induced by crotonaldehyde. As the principal participant in the crotonaldehyde-induced survival pathway of HepG2 cells to resist apoptosis, HO-1 may represent a therapeutic target in crotonaldehyde-induced resistance to tumors. The lignan tigloylgomisin H (TGH) is extracted from Schisandra chinensis fruit. is an additional botanical product that has been illustrated to have the capability of activating and translocating Nrf2 into the nucleus (222). In Hepa1c1c7 and BPrc1 cell lines, TGH triggered a phase II detoxifying enzyme, NQO1. Thus, TGH may function as a prospective preventive agent against HCC.

**Figure 4:**
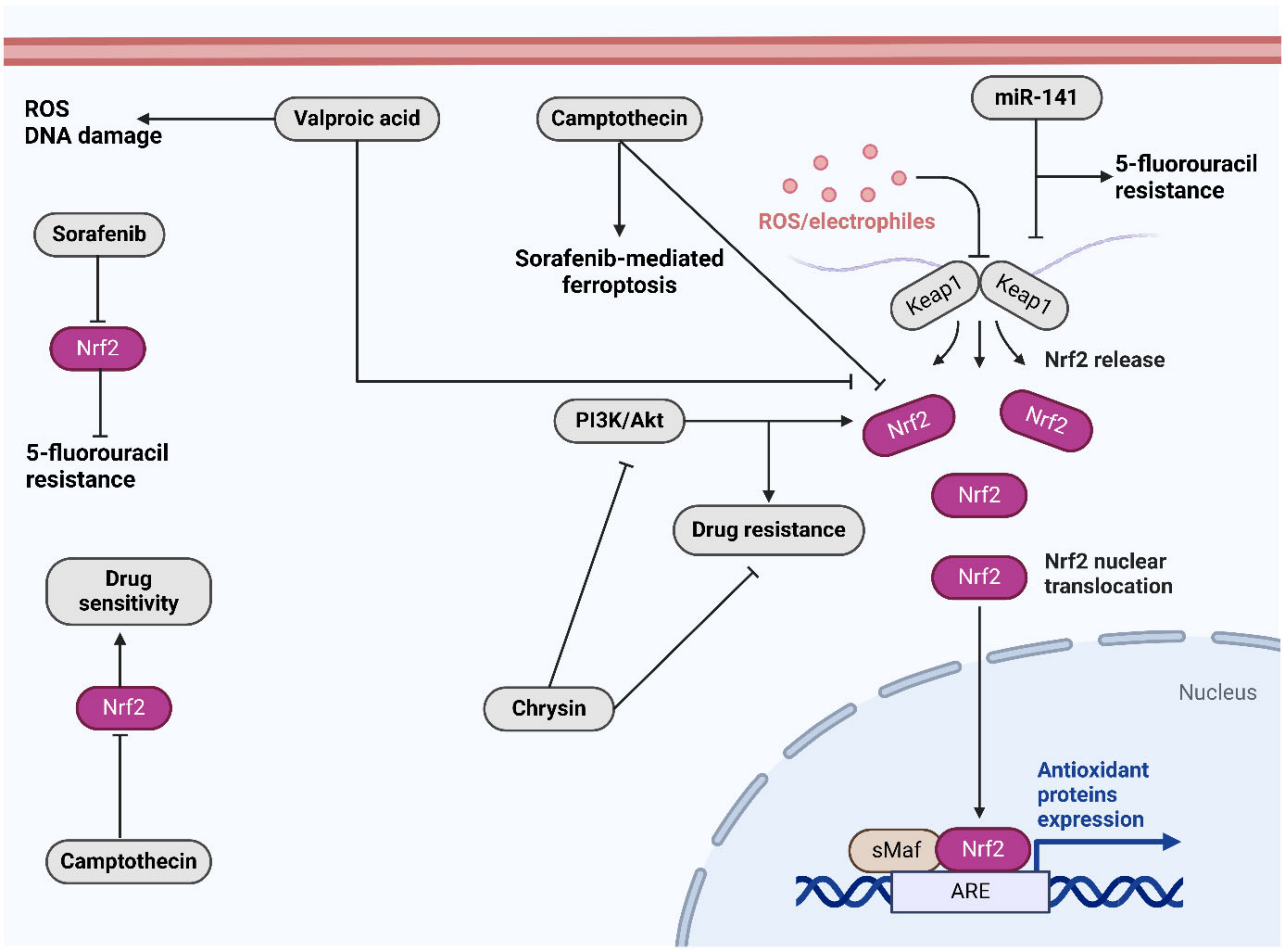
The role of Nrf2 in drug resistance and its regulation by therapeutic compounds. Sorafenib downregulates Nrf2 to overcome 5-flourouracil resistance. Moreover, camptothecin downregulates Nrf2 to increase drug sensitivity. Chrysin suppresses PI3K/Akt axis to suppress Nrf2. Created with BioRender.com.

The fruit of the Punica granatum pomegranate is considered a superfruit due to its substantial content of polyphenolic compounds and powerful antioxidant attributes (223). In rodents, The antioxidant effects of pomegranate juice were found to be systemic (224). Pomegranate emulsion (PE) inhibits DENA-induced rodent hepatocarcinogenesis, which mimics human HCC (225). By means of Nrf2 activation, In rats that were treated with DENA, the PE’s active components caused the activation of enzymes that detoxify carcinogens and antioxidants in the liver. Protein and lipid oxidation is slowed down by PE. Hence, the presence of active constituents in the PE may serve as preventative agents against cancer. Camptotheca acuminata is known to yield camptothecin, a planar pentacyclic quinolone alkaloid with antitumor properties (226). Camptothecin functions as a DNA topoisomerase I poison by forming a stable complex to DNA topoisomerase type I. This complex inhibits cellular proliferation by impeding the DNA replication reactions of cleavage and relegation. Camptothecin has been recognized serving as an effective inhibitor of the Nrf2 gene in recent years. Camptothecin inhibited the expression of Nrf2 in xenograft models and advanced liver cancer cells (HepG2 and SMMC-7221) additionally, it sensitized the cells to a range of chemotherapeutic agents (227). Combinatorial therapy may be expanded in malignancies with elevated Nrf2 expression by camptothecin. All of these phytochemicals and additional molecules have the ability to modulate Nrf2 activation in HCC by targeting distinct molecules. **Figure 4** shows the function of Nrf2 in cancer drug resistance and its regulation by therapeutic compounds. **Table 1** summarizes the role of Nrf2 in the regulation of HCC progression.

**Table 1:** Evaluating the function of Nrf2 in the regulation of HCC progression.

Molecular pathway	Remark	Reference
pSmad3C/3L and Nrf2/HO-1	Astragaloside IV disrupts cancer progression through reducing fibrosis by upregulating pSmad3C, pNrf2, HO-1, and NQO1 and downregulating pSmad2C, pSmad2L, pSmad3L, PAI-1, and α-SMA	(228)
FNDC5	FNDC5 stimulates PI3K/Akt/Nrf2 axis to mediate sorafenib resistance	(229)
Nrf2	Chlorogenic Acid decreases apoptosis and DNA damage through Nrf2 upregulation	(230)
p62-Keap1-Nrf2	Arenobufagin mediates autophagy-related ferroptosis	(231)
Nrf2	The SUMOylation of Nrf2 increases serine synthesis and increases cancer progression	(232)
Nrf2/HO-1 and TGF-β1/Smad3	Astragaloside IV disrupts metastasis through upregulating Nrf2/pNrf2, HO-1, pSmad3C, and p21 and downregulating pSmad3L and c-Myc	(233)
CPLX2	CPLX2 enhances Nrf2 levels to reduce apoptosis and ferroptosis	(234)
p62-Keap1-Nrf2	Metformin and sorafenib combination can downregulate Nrf2 to enhance ferroptosis	(235)
ALDH2	ALDH2 prevents immune evasion through ROS/Nrf2-induced autophagy	(236)
Nrf2	Camptothecin downregulates Nrf2 to suppress angiogenesis and metastasis	(237)
Nrf2/ARE	Camptothecin downregulates Nrf2/ARE axis to enhance sorafenib sensitivity	(238)
GSTZ1	Loss of GSTZ1 stimulates Nrf2 to enhance proliferation	(239)
NRF2/SHH	Enhancement in tumor-initiating cell lineage and chemoresistance	(120)
CDCA2	CDCA2 supports against oxidative damage through inducing BRCA1/Nrf2 axis	(240)
Nrf2	Enhancement in stemness, metastasis and ABC transporter gene expression	(241)
Nrf2/EMT	Regulation of cancer metastasis	(242)
SLC27A5	Loss of SLC27A5 stimulates Nrf2/TXNRD1 axis through lipid peroxidation	(243)
Nrf2	Nrf2-siRNA can suppress HIF-1α/HSP70 to enhance drug sensitivity	(244)
Nrf2/MAPK	Oxygen therapy controls Nrf2/MAPK axis to promote apoptosis mediated selenium compounds	(245)
TRIM25	TRIM25 stimulates Nrf2 to enhance survival and growth of HCC	(184)
NOX4	NOX2 upregulation increases mitochondrial ROS levels to enhance tumorigenesis and mediate mitophagy by Nrf2/PINK1 axis	(246)
Nrf2	Upregulated Nrf2 promotes HIF-1α stability to mediate pseudohypoxia	(188)
UBR7	UBR7 impairs cancer progression through regulation of Keap1/Nrf2/Bach1/HK2 and glycolysis	(247)
Nrf2	Nrf2 mediates metabolism of Acetyl-CoA and promotes tumorigenesis	(182)
IGF2BP3/NRF2	IGF2BP3 increases Nrf2 stability to prevent ferroptosis	(248)
SOCS1	Loss of SOCS1 promotes Nrf2 levels to mediate tumorigenesis	(197)
c-Myc	c-Myc increases GOT1 and Nrf2 levels to reduce ferroptosis	(249)

## 8 Nrf2 in immune evasion: implications for hepatocellular carcinoma

Extensive research has shown that activating Nrf2 in lung adenocarcinoma weakens the immune system’s response to tumors and decreases chemotherapy effectiveness (250). Patients with LOF mutations in Keap1 or activating mutations in Nrf2 typically have worse outcomes when undergoing therapy with checkpoint inhibitors (251, 252). Similar patterns have been observed in a broad range of cancers, according to comprehensive cancer studies (253, 254). Notably, mutations in Keap1 are linked to a higher tumor mutational burden and elevated PD-L1 levels, which are often associated with better outcomes following checkpoint inhibitor treatments (255). The mechanisms of resistance linked to these genetic alterations are not well understood, with limited research focusing on the immune environments of Keap1-mutant tumors. Initial studies by Kadara and others (256) suggest that tumors with Keap1 mutations show increased numbers of CD57+ and granzyme B+ cells around the tumor, indicating enhanced NK cell presence. In experiments with a Keap1flox/flox; Ptenflox/flox (K1P) mouse model, Best and associates (257) observed a decrease in NK, B, and T cells in the lungs of mice with tumors compared to healthy controls, alongside a reduction in the growth of CD11c+ immune populations. Nrf2 also impacts how specific immune cells function. Kobayashi and team (258) found that Nrf2 suppresses the production of Il6 and Il1β in M1 macrophages triggered by LPS. Similarly, Thimmulappa and team (259) discovered that peritoneal neutrophils deficient in Nrf2 produce lower levels of IL6 and TNFα in response to LPS. Additionally, Nrf2 affects cytokine production within tumors. It has been shown to facilitate IL33 release, a cytokine implicated in promoting tumor progression by encouraging protumor macrophage buildup in skin squamous cell carcinoma (260). Regarding Il11, research by Nishina and colleagues (261) demonstrated that Nrf2 activation in a mouse colorectal cell line increases Il11 transcription. Kitamura and team (113) linked higher IL11 protein levels with Nrf2 expression in human breast cancer and found that Keap1-null MEFs in three-dimensional culture elevate Il11, which crucially impacts tumor engraftment and is reduced by Il11 knockout. These findings highlight Nrf2’s significant role in controlling cytokine levels, which can profoundly affect cancer development. Nrf2’s role in immune evasion in HCC primarily hinges on its capacity to modulate the tumor microenvironment in ways that inhibit effective immune surveillance and response. Through its regulation of antioxidative stress responses, Nrf2 enhances the survival and proliferation of HCC cells under oxidative stress, a common feature of tumor environments. This not only helps tumor cells cope with the hostile conditions but also makes them less susceptible to immune-mediated destruction. Moreover, Nrf2 drives the expression of various cytoprotective genes that produce enzymes and proteins to detoxify potential immune mediators such as reactive oxygen species (ROS), which are utilized by immune cells as part of their tumor-killing function. The result is a reduced efficacy of immune cells like natural killer (NK) cells and cytotoxic T lymphocytes, which rely on inducing oxidative stress in tumor cells to promote apoptosis. In addition to direct protection against immune cell functions, Nrf2 also influences the recruitment and function of immunosuppressive cells within the tumor microenvironment. It upregulates factors that attract regulatory T cells (Tregs) and myeloid-derived suppressor cells (MDSCs), both known for their roles in immune suppression and their ability to promote tumor growth. Nrf2’s activation increases the levels of immunosuppressive cytokines such as IL-10 and TGF-β, further promoting an environment conducive to tumor progression and resistance to immune checkpoint blockade therapies. This modulation of the cytokine milieu not only hampers the cytotoxic activity of immune effector cells but also enhances the expression of checkpoint molecules like PD-L1 on tumor cells, strengthening their ability to evade immune surveillance. Through these pathways, Nrf2 contributes to a fortified barrier against immune system attacks, enabling HCC cells to thrive and expand unchallenged by the body’s natural defenses.

## 9 Nrf2 signaling in combination cancer therapy: perspectives on hepatocellular carcinoma

Regarding the aggressive behavior of HCC cells, various studies have focused on the introduction of multiple strategies in HCC therapy. Radiotherapy has been used significantly for the treatment of HCC. However, different studies have shown the radioresistance in HCC. UBET2 and its interaction with H2AX can increase CHK1 levels to mediate radioresistance in HCC (262). Moreover, increase in DNA repair mediated by TKT/PARP1 axis can mediate radioresistance in HCC (263). The c-Myc (264), RNF6 (265) and NEAT1 (266, 267) are other factors capable of regulating radioresistance in HCC. On the other hand, the increasing evidences have shown the capacity of Nrf2 in the induction of radioresistance in human cancers (268–270). Regarding this, it is suggested that future studies focus on the application of Nrf2 inhibitors along with radiotherapy to exert synergistic impact in HCC therapy and promote radiosensitivity. On the other hand, chemoresistance also commonly occurs in HCC (271–273). The upregulation of lncRNA SNHG16 in HCC can impair the growth and drug resistance through sponging miR-93 (274). Moreover, CMTM6 reduces the p21 ubiquitination to disrupt the proliferation and overcome chemoresistance in HCC (275). The studies have demonstrated the potential of Nrf2 in the regulation of chemoresistance discussed in previous section and therefore, the application of Nrf2 inhibitors along with chemotherapy agents can improve the efficacy in HCC elimination. Immunotherapy is another therapeutic strategy for HCC that has been compromised by the emergence of immune evasion. HERC2-mediated STAT3 upregulation (276), PRDM1/BLIMP1 (277), DGKG (278) and circulating tumor cells (279) are among the major factors contributing to the immune evasion. The mitochondrial TSPO reduces ferroptosis and enhances immune evasion in HCC through enhancing Nrf2 levels (280). According to this, the co-application of Nrf2 inhibitors and immunotherapy can impair immune evasion and promote immune reactions.

## 10 Dual function of Nrf2: perspectives in hepatocellular carcinoma therapy

Nrf2 is a transcription factor that plays a pivotal role in cellular defense against oxidative stress and toxin-induced damage by regulating the expression of antioxidant proteins that protect against oxidative damage triggered by injury and inflammation. In the context of HCC, Nrf2’s role is complex, exhibiting both anti-cancer and pro-carcinogenic activities, which hinge on the balance of cellular contexts and disease stages. In its anti-cancer role, Nrf2 contributes to hepatocyte protection by enhancing the cellular antioxidant capacity. Under normal physiological conditions, Nrf2 is kept in the cytoplasm by Kelch-like ECH-associated protein 1 (Keap1) and targeted for ubiquitination and proteasomal degradation. However, in response to oxidative stress, Nrf2 dissociates from Keap1, translocates to the nucleus, and binds to antioxidant response elements (ARE) in the DNA. This binding induces the transcription of various detoxifying and antioxidant enzymes, such as glutathione S-transferase, heme oxygenase-1, and NAD(P)H quinone oxidoreductase 1. By elevating the levels of these molecules, Nrf2 helps mitigate the effects of oxidative stress and decreases the risk of mutations and other oncogenic processes, thus providing a protective mechanism against the initiation of cancer, including HCC. Conversely, the carcinogenic role of Nrf2 in HCC is linked to its ability to promote cancer cell survival under oxidative stress conditions, which are common in tumor microenvironments. When Nrf2 activity becomes constitutively upregulated, often through mutations in the Keap1 gene or through the gain-of-function mutations within Nrf2 itself, it can lead to an abnormal accumulation of Nrf2 in the nucleus. This results in the persistent activation of target genes that not only protect against oxidative stress but also promote cell proliferation and tumor progression. The sustained high levels of antioxidants and detoxifying enzymes can provide cancer cells with a growth advantage and resistance to chemotherapeutic agents, ultimately supporting tumor growth and contributing to poor prognosis in HCC patients. Furthermore, the interplay between Nrf2 and other molecular pathways exacerbates its carcinogenic potential. Studies have shown that Nrf2 can interact with pathways that regulate cell proliferation and apoptosis, such as the PI3K/Akt pathway, thereby enhancing cancer cell growth and survival. Nrf2’s upregulation has been associated with increased expression of anti-apoptotic proteins and downregulation of pro-apoptotic proteins, tilting the balance towards cell survival. Moreover, Nrf2 can induce the expression of metabolic enzymes and transporters that contribute to the efflux and decreased effectiveness of chemotherapeutic drugs, fostering chemoresistance in HCC cells. This aspect of Nrf2’s function is particularly detrimental, as it not only facilitates tumor progression by protecting malignant cells from oxidative damage but also enables these cells to evade the cytotoxic effects of treatment, posing significant challenges for the management of HCC.

## 11 Conclusion

Inhibitors of Nrf2 in conjunction with standard anti-neoplastic treatments may represent a viable strategy for combating HCC. Nrf2 facilitates the development and formation of multidrug resistance (MDR) mechanisms in cancer cells, such as MRP family members, detoxifying enzymes, anti-oxidative stress genes, and enhanced anti-apoptosis capabilities. The complexity of target genes and regulation mechanisms, the lack of fully illuminated cross-talk between the Nrf2/ARE signaling pathway and other signal conduction pathways, and the inadequately understood roles of Nrf2 and Keap1 *in vivo* are a few of the obstacles that must be surmounted. Furthermore, there is an absence of documentation regarding the utilization of Nrf2 inhibitors as adjuvant sensitizers in clinical trials. HCC is primarily characterized by increased expression of Nrf2, which is the key factor in HCC. Vascularization, drug resistance, and cellular proliferation are all outcomes of aberrant Nrf2 signaling. By modulating Nrf2 expression, natural phytochemicals can reverse chemotherapeutic resistance in HCCs and induce anticancer effects. Therapeutic opportunities against HCC may be created by targeting miRs with miRs. As prospective treatments for HCC, cancer pharmacologists are actively engaged in the identification of potent and safe Nrf2 inhibitors. Additionally, We need more studies and molecular profiling to understand how Keap1 and Nrf2 regulate epigenetics in HCC.

The dual function of Nrf2 between HCC and normal liver tissues has provided a challenge for the development of therapeutics affecting Nrf2 in HCC, while maintaining its protective function in liver tissue. In respect to this, a number of factors should be considered during the development of Nrf2 inhibitors. It is essential to understand the differential pathways and context-specific activation of Nrf2. The induction of Nrf2 in the liver tissues is mainly based on oxidative damage to increase antioxidant defense system. However, Nrf2 mutation or KEAP1 downregulation in HCC can enhance the tumorigenesis and drug resistance along with immune evasion. Therefore, the specific Nrf2 inhibitors should be developed based on preserving the protective function of Nrf2 in the liver tissues, while minimizing its impact in HCC cells. Moreover, there should be some comprehensive studies evaluating the specific structural differences and molecular alterations of Nrf2 in the liver tissue and HCC. The application of high-throughput screening of the small molecules and computational drug design can be beneficial in the selective targeting of Nrf2 mutant or disrupting the Nrf2 and KEAP1 complex in HCC. The combination therapy is also suggested in this case that Nrf2 and other pathways responsible for HCC survival are targeted not in normal hepatocytes. In this case, the Nrf2 inhibitors can be used along with factors affecting HCC survival to finally improve the selectivity and therapeutic index in HCC elimination. In order to provide a milestone in the regulation of Nrf2, it is suggested to utilize RNAi and CRISPR technologies to specifically target Nrf2 mutants in HCC therapy lacking impact on normal hepatocytes. An approach that has been ignored is the application of nanoparticles. In the recent years, nanoparticles have been considered as potent regulators of Nrf2 in disease therapy (281–284). Therefore, an idea can be the development of stimuli-responsive nanoparticles capable of releasing Nrf2 inhibitors in response to changes in the tumor microenvironment to provide specific delivery to HCC cells not hepatocytes.

Nrf2 plays a critical role in developing chemoresistance in HCC. It enhances the expression of genes that help detoxify harmful substances and expel drugs from the cells, thus reducing their efficacy. The activation of the Nrf2/ARE pathway increases the expression of antioxidant proteins that protect cancer cells from the oxidative stress induced by chemotherapy, thereby promoting survival and proliferation. Nrf2 impacts several key signaling pathways associated with cell survival, proliferation, and metastasis. It interacts with pathways like PI3K/Akt, which are crucial for cell survival and growth. By modulating these pathways, Nrf2 helps in maintaining a pro-survival environment in HCC cells. Moreover, Nrf2’s interaction with other molecules such as Keap1 also modulates its activity and stability, further influencing cancer progression and resistance mechanisms. The sustained activation of Nrf2 not only protects cells from oxidative damage but also promotes oncogenic activities by supporting rapid cell growth and inhibiting apoptosis. This dual role of Nrf2, especially its ability to enhance the survival of transformed cells in the hypoxic and oxidative stress-rich environment of tumors, contributes to the aggressiveness of HCC. Nrf2 also plays a role in immune evasion by modulating the immune microenvironment of HCC. It affects the expression of cytokines and other mediators that can alter immune surveillance, thus facilitating tumor growth and progression by dampening the immune response.

The clinical applications of targeting Nrf2 in HCC involve exploring its dual roles in cancer cell metabolism, oxidative stress response, and drug resistance. Nrf2 helps cells cope with oxidative stress by upregulating AREs, which can be beneficial in normal cells but problematic in cancer cells where it promotes survival and growth. Inhibiting Nrf2 in HCC could reduce the cancer cells’ ability to resist oxidative stress induced by cellular metabolism or therapeutic interventions. Nrf2 contributes to chemoresistance in HCC by enhancing the expression of detoxifying enzymes and drug efflux pumps. Inhibitors of Nrf2 or disruption of its signaling pathway could potentially enhance the efficacy of chemotherapeutics by making cancer cells more susceptible to oxidative damage and apoptosis. Given its role in antioxidant defense and drug resistance, Nrf2 could serve as a biomarker for predicting the aggressiveness of HCC or the likelihood of response to certain therapies, particularly those inducing oxidative stress in cancer cells. Nrf2’s function as both a protector against oxidative damage in normal cells and a promoter of survival in cancer cells complicates its targeting. Complete inhibition could lead to unwanted toxicity and exacerbation of liver damage in normal or non-tumor liver tissue. Cancer cells might develop compensatory mechanisms that bypass Nrf2 inhibition, such as activating alternative pathways for detoxification and survival, thereby reducing the effectiveness of Nrf2-targeted therapies. Genetic variations in Nrf2 or its regulatory proteins (like Keap1) among patients might lead to different responses to Nrf2-targeted treatments, necessitating personalized approaches in therapy and dosing. Developing selective Nrf2 inhibitors that target only its cancer-promoting activities while preserving its normal cellular protective functions. This could involve targeting specific domains of Nrf2 or its interaction with other proteins like Keap1. Combining Nrf2 inhibitors with other treatments such as chemotherapy, targeted therapy, or immunotherapy to enhance overall treatment efficacy and possibly prevent the development of resistance. Utilizing nanoparticle-based delivery systems to target Nrf2 inhibitors specifically to tumor cells, minimizing systemic exposure and potential side effects. Further research into the genetic and epigenetic regulation of Nrf2 in HCC could unveil new therapeutic targets within the Nrf2 signaling pathway and help in the development of more effective, personalized treatment strategies.
